# Socioeconomic status, social capital, health risk behaviors, and health-related quality of life among Chinese older adults

**DOI:** 10.1186/s12955-020-01540-8

**Published:** 2020-08-28

**Authors:** Ying Yang, Shizhen Wang, Lei Chen, Mi Luo, Lina Xue, Dan Cui, Zongfu Mao

**Affiliations:** 1 Global Health Institute, Wuhan University, 115# Donghu Road, Wuhan, 430071 China; 2 School of Health Sciences, Wuhan University, 115# Donghu Road, Wuhan, 430071 China

**Keywords:** Social capital, Health risk behaviors, Health behavior, Quality of life

## Abstract

**Background:**

There is limited knowledge on the mediating role of different health risk behavior on the relationship between social capital, socioeconomic status (SES), and health-related quality of life (HRQoL) in Chinese older adults. We conducted this study to (a) investigate the condition of health risk behaviors of the Chinese elderly, and (b) assess the relationship between SES, social capital, health risk behaviors, and HRQoL.

**Methods:**

A sample of 4868 adults aged 60 years and older were included in this study, from the China’s Health-related Quality of Life Survey for Older Adults 2018. Participants’ demographic characteristics, SES (education level, family income), health risk behaviors (smoking, alcohol consumption, physical inactivity, unhealthy dietary behavior, unhealthy weight, and sleep disorder) were collected. Social capital and HRQoL were assessed by the 16-item Personal Social Capital Scale (PSCS-16) and WHOQOL-Old, respectively. Structural equation modeling (SEM) was applied to examine the associations between variables.

**Results:**

The proportion of smoking, alcohol consumption, physical inactivity, unhealthy dietary behavior, unhealthy weight, and sleep disorder were 32.1, 36.3, 62.5, 45.7, 31.8, and 45.5%, respectively. Significant differences were observed in education level, family income, and social capital between elderly individuals with and without each of the six health risk behavior (all *p*-values < 0.05). Elderly individuals who reported smoking, physical inactivity, unhealthy dietary behavior, and sleep disorder had significantly lower HRQoL than those without these unhealthy behaviors (all *p*-values < 0.05). SEM analysis showed that SES and social capital positively associated with alcohol consumption. Social capital negatively associated with smoking, physical inactivity, unhealthy dietary behavior, and sleep disorder. SES negatively associated with smoking, physical inactivity, unhealthy dietary behavior, unhealthy weight, and sleep disorder. Smoking, physical inactivity, unhealthy dietary behavior, and sleep disorder correlated with poorer HRQoL.

**Conclusions:**

Chinese older adults demonstrated a high incidence of health risk behaviors, especially for physical inactivity (62.5%) and unhealthy dietary behavior (45.7%). Social capital and SES were correlated with the elderly’s HRQoL, as well as with the health risk behaviors. Health risk behaviors played potential mediating role on the relationship between social capital, SES, and HRQoL in Chinese older adults.

## Background

By the end of 2018, 249 million elderly residents are aged 60 years and older in China which account for 17.9% of the Chinese population [1]. In China, nearly 75% of the elderly suffer from chronic diseases and 40 million elderly people live with disability or partially disability [2–4]. Chinese elderly are undergoing unoptimistic health condition.

Daily behavioral lifestyles, such as smoking, drinking, physical activity, body mass index (BMI), dietary behavior, seat belt use, sleep impairment, are correlated with older adults’ health [5, 6]. About 60% of the health influence factors are related to individual behavior and lifestyle, and the effective control of health risk behaviors can prevent many disease [2, 7]. The identification of the impact of health risk factors on elderly health outcomes will help to better target health promotion [2].

Social capital is an intangible resource constructed by social relationship, which has effects on health promotion [8–10]. The components of social capital, i.e. social networks, social participation, and trust, are related to self-rated health, mental health, and depressive symptom of elderly people [11]. Previous studies explored the impact and its pathways of social capital on individual health [12–16]. Huang et al. [17] and Chen et al. [18] indicated that social capital might indirectly affect the elderly’s physical and mental health through their behaviors and lifestyle. However, several researchers reported the inconsistent results on the relationship between social capital and different health risk behaviors in Chinese older adults [19, 20]. Also, the mediating role of difference health risk behaviors on the relationship between social capital and quality of life are not yet clarified in the elderly population.

In addition, socioeconomic status (SES) is widely considered to associate with individuals’ behavior and health [21–24]. Wang et al. [25] mentioned that health-related behaviors could mediate the relationship between SES and elderly health. The relationship between SES and different health-related behaviors are different. For example, people with Low SES are more likely to report physical inactivity because of poor access to sports resources, but they are less likely to develop overweight or obesity due to relatively poor living and working conditions. Thus, the mediating role of different health-related behaviors on the relationship between SES and elderly health are worth exploring and need to be clarified.

In response to the above gaps, structural equation model (SEM) was employed to analyze relationships among SES, social capital, health risk behaviors, and health-related quality of life (HRQoL). The objectives of this study were to (a) describe the condition of health risk behaviors of Chinese older adults, and (b) examine the associations between SES, social capital, health risk behaviors, and HRQoL.

## Method

### Study population

This study used data from China’s Health-related Quality of Life Survey for Older Adults 2018 (CHRQLS-OA 2018). The survey employed a cross-sectional design and collected data using self-completion questionnaires. In China, the CHRQLS-OA 2018 was organized and conducted by the Global Health Institute of Wuhan University during the Spring Festival in 2018. The survey aimed to collect data on the socio-ecological factors and health status of the elderly in China. All participants were aged 60 years and older. Using a convenience sampling strategy, a general database containing 5442 valid samples was finally established. The survey was conducted both online and offline during the Spring Festival, when the population is most evenly distributed in China. The response rate for the offline survey was 85.26%. Details of the CHRQLS-OA 2018 are available in a previous work done by our team [26].

In this study, we aimed to assess the association between SES, social capital, health risk behaviors, and HRQoL, thus we excluded subjects with no relevant information (*n* = 574). Finally, 4868 individuals aged 60 years and older were included in the analysis.

### Conceptual framework

The conceptual framework of this study was adapted from the WHO conceptual framework for social determinants of health [27] (see supplementary figure 1). It was expected a priori that structural determinants, including poor socioeconomic status (low education level, low family income), low social capital would predict intermediary determinants (i.e. health risk behaviors in this study). Structural and intermediary determinants were also expected to predict worse HRQoL. In addition, the intermediary determinants would mediate the relationship between structural determinants and HRQoL.

### Variables

#### Demographic characteristics

Participants’ demographic characteristics were collected, including age, gender, marital status, and place of residence. Marital status was dichotomized into married and others (single, divorced, and widowed). Place of residence was divided into urban areas and rural areas.

#### Socioeconomic status

Socioeconomic status was a latent variable measured by two indicators: education level and family per-capita annual income (CHY). Education level was categorized as: 1 = below primary school, 2 = primary school, 3 = middle school or high school, and 4 = college and above. Family per-capita annual income (CHY) was divided into four groups: 1 = < 15,000, 2 = 15,000-30,000, 3 = 30,000-45,000, and 4 = ≥ 45,000.

#### Social capital

Social capital was a latent variable using the dimensions of the 16-item Personal Social Capital Scale (PSCS-16) instrument as indicators [28]. PSCS-16 contains two subscales: bonding social capital (8 items) and bridging social capital (8 items). Bonding social capital refers to how well a person is embedded within their various networks of different types of people (e.g., family members, friends, former colleagues), and bridging social capital refers to how well a person is embedded within different types of social organizations [28]. Each item applied a 5-point Likert scale: 1 (a few), 2 (less than average), 3 (average), 4 (more than average), and 5 (a lot). The total score of PSCS-16 is obtained by adding up the scores of the items and can vary from 16 to 80. A higher PSCS-16 score indicates greater social capital.

#### Health risk behaviors

Six health risk behaviors were included in this study: (1) smoking, current smokers were categorized as a smoking group; (2) alcohol consumption was defined as drinking frequency ≥ 1 time per week; (3) physical inactivity, individuals who did not meet the standard set by the Chinese Center for Disease Control and Prevention (CDC), i.e. doing exercise more than three times per week and at least 30 min per time, were identified as physical inactivity; (4) unhealthy dietary behavior, individuals who self-reported skipping breakfast or having an unbalanced diet such as an insufficient intake of vegetables and fruit were identified as having unhealthy dietary behavior; (5) unhealthy weight was identified as body mass index (BMI) > 26.9 kg/m^2^ or < 20 kg/m^2^ based on the BMI criteria for Chinese elderly from Chinese Nutrition Society (CNS) [29]. BMI was calculated through self-reported height and weight; (6) Sleep disorder was assessed by the question “Do you have any sleep problems, such as insomnia, dreaminess or unstable sleep, fitful sleep, hypersomnia? (yes/no)” The response of “yes” represented having sleep disorder.

#### Health-related quality of life

The outcome of HRQoL was assessed using the WHOQOL-Old [30]. WHOQOL-Old is a well-developed instrument with adequate reliability and validity, and has been widely used to assess HRQoL in many countries [31]. The WHOQOL-Old contains 24 items distributed into six subscales: sensory abilities; autonomy; past, present, and future activities; social participation; death and dying; and intimacy. Each item was scored on a Likert-type scale ranging from 1 to 5, with two subscales (sensory abilities, death and dying) applied reverse scoring. The total score ranges from 24 to 120, with higher score indicates better HRQoL. HRQoL was identified as a latent variable using the scores of each subscale as indicators.

### Statistical analysis

Descriptive statistics were conducted including means and standard deviations (*SD*) (continuous variables), frequency and percentage (categorical variables). Chi-square test and *t*-test were used to compare the differences of SES, social capital, and HRQoL between participants with and without a certain health risk behavior. Confirmatory factor analysis (CFA) was applied to evaluate the measurement model involving three latent variables (socioeconomic status, social capital, and HRQoL). Structural equation modeling was used to verify the direct and indirect relationships between observed and latent variables according to the conceptual framework. Parameters were estimated by the maximum-likelihood method. The evaluation of the model fit was based on the following criteria: standardized root-mean-square residual (SRMR) ≤ 0.08, root-mean-square error of approximation (RMSEA) ≤ 0.08, goodness of fit index (GFI) ≥ 0.90, comparative fit index (CFI) ≥ 0.90, normed fit index (NFI) ≥ 0.90 [32]. Data analysis was conducted using IBM SPSS Statistics 22.0 and IBM SPSS AMOS 24.0 software. In all analyses, a *p*-value of < 0.05 was considered statistically significant.

## Results

### Descriptive statistics

Table 1 summaries the results of descriptive statistics. A total of 4868 elderly people with an average age of 71.0 years (*SD* = 7.8) participated in this study. Among the participants, 49.5% (*n* = 2408) were male, 67.6% (*n* = 3292) were married, 78.0% (*n* = 3797) lived in rural areas, 66.4% (*n* = 3232) had an education level of primary school and below, 36.0% (*n* = 1751) reported a family per-capita annual income of less than 15,000 CHY. The overall score of social capital and HRQoL were 41.9 (*SD* = 14.5) and 77.3 (*SD* = 12.4). The proportion of smoking, alcohol consumption, physical inactivity, unhealthy dietary behavior, unhealthy weight, and sleep disorder were 32.1, 36.3, 62.5, 45.7, 31.8, and 45.5%, respectively.

**Table 1:** Participants’ demographic characteristics, socioeconomic status, social capital, health risk behaviors, and health-related quality of life (HRQoL)

Variables	***N*** = 4868
Age, mean ± *SD*	71.0 ± 7.8
Gender (1 = Male, 0 = Female)	2408 (49.5)
Marital status (1 = Married, 0 = Others)	3292 (67.6)
Place of residence (1 = Rural, 0 = Urban)	3797 (78.0)
**Socioeconomic status**	
Education level	
< Primary school	1971 (40.5)
Primary school	1261 (25.9)
Middle/high school	1283 (26.4)
≥ College	353 (7.3)
Family per-capita annual income (CHY)	
≤ 15,000	1751 (36.0)
15,000-30,000	1217 (25.0)
30,000-45,000	936 (19.2)
> 45,000	964 (19.8)
**Health risk behaviors**	
Smoking (1 = Yes, 0 = No)	1563 (32.1)
Alcohol consumption (1 = Yes, 0 = No)	1769 (36.3)
Physical inactivity (1 = Yes, 0 = No)	3044 (62.5)
Unhealthy dietary behavior (1 = Yes, 0 = No)	2226 (45.7)
Unhealthy weight (1 = Yes, 0 = No)	1549 (31.8)
Sleep disorder (1 = Yes, 0 = No)	2214 (45.5)
**Social capital**	
Overall social capital, mean ± *SD*	41.9 ± 14.5
Bonding social capital, mean ± *SD*	22.7 ± 7.2
Bridging social capital, mean ± *SD*	19.2 ± 8.2
**HRQoL**	
Overall HRQoL, mean ± *SD*	77.3 ± 12.4
Sensory abilities, mean ± *SD*	12.8 ± 2.9
Autonomy, mean ± *SD*	13.6 ± 3.3
Past, present and future activities, mean ± *SD*	13.0 ± 3.0
Social participation, mean ± *SD*	13.0 ± 2.9
Death and dying, mean ± *SD*	12.1 ± 3.3
Intimacy, mean ± *SD*	12.8 ± 3.3

Continuous variables are presented as mean and standard deviation (*SD*), categorical variables are presented as frequency (*n*) and percentage (%). For binary variables, the frequency *(n*) and percentage (%) of observations coded 1 are presented

### Univariate analysis

We compared the differences of SES, social capital, and HRQoL between participants with and without a certain health risk behavior (Table 2). Participants with different education level and income level had significantly different proportion for all six health risk behaviors (all *p*-values < 0.01). Significantly lower social capital score was observed in elderly individuals with the behavior of smoking (*t* = 3.29, *p* < 0.01), physical inactivity (*t* = 15.48, *p* < 0.001), unhealthy dietary behavior (*t* = 15.50, *p* < 0.001), unhealthy weight (*t* = 2.59, *p* < 0.05), and sleep disorder (*t* = 14.49, *p* < 0.001) than individuals without these behaviors. Participants with the behavior of alcohol consumption had significantly higher social capital score than those without the behavior (*t* = − 3.05, *p* < 0.01). Significantly lower HRQoL score was observed in participants with the behavior of smoking (*t* = 6.03, *p* < 0.001), physical inactivity (*t* = 25.83, *p* < 0.001), unhealthy dietary behavior (*t* = 17.15, *p* < 0.001), and sleep disorder (*t* = 17.13, *p* < 0.001) than individuals without these behaviors.

**Table 2:** Socioeconomic status, social capital, and HRQoL in participants with or without a certain health risk behavior

Variables	Smoking		Alcohol consumption		Physical inactivity		Unhealthy dietary behavior		Unhealthy weight		Sleep disorder	
	Yes	No	Yes	No	Yes	No	Yes	No	Yes	No	Yes	No
**Education level**												
< Primary school	593 (30.1)	1378 (69.9)	644 (32.7)	1327 (67.3)	1351 (68.5)	620 (31.5)	1045 (53.0)	926 (47.0)	642 (32.6)	1329 (67.4)	927 (47.0)	1044 (53.0)
Primary school	377 (29.9)	884 (70.1)	443 (35.1)	818 (64.9)	777 (61.6)	484 (38.4)	541 (42.9)	720 (57.1)	436 (34.6)	825 (65.4)	547 (43.4)	714 (56.6)
Middle/high school	465 (36.2)	818 (63.8)	524 (40.8)	759 (59.2)	753 (58.7)	530 (41.3)	505 (39.4)	778 (60.6)	371 (28.9)	912 (71.1)	603 (47.0)	680 (53.0)
≥ College	128 (36.3)	225 (63.7)	158 (44.8)	195 (55.2)	163 (46.2)	190 (53.8)	135 (38.2)	218 (61.8)	100 (28.3)	253 (71.7)	137 (38.8)	216 (61.2)
*χ*^*2*^	19.38^***^		34.30^***^		79.24^***^		75.20^***^		11.90^**^		11.69^**^	
**Income level**												
< 15,000 CHY	612 (35.0)	1139 (65.0)	584 (33.4)	1167 (66.6)	1389 (79.3)	362 (20.7)	885 (50.5)	866 (49.5)	676 (38.6)	1075 (61.4)	966 (55.2)	785 (44.8)
15,000–30,000 CHY	406 (33.4)	811 (66.6)	445 (36.6)	772 (63.4)	870 (71.5)	347 (28.5)	620 (50.9)	597 (49.1)	375 (30.8)	842 (69.2)	587 (48.2)	630 (51.8)
30,000–45,000 CHY	278 (29.7)	658 (70.3)	351 (37.5)	585 (62.5)	410 (43.8)	526 (56.2)	371 (39.6)	565 (60.4)	258 (27.6)	678 (72.4)	366 (39.1)	570 (60.9)
≥ 45,000 CHY	267 (27.7)	697 (72.3)	389 (40.4)	575 (59.6)	375 (38.9)	589 (61.1)	350 (36.3)	614 (63.7)	240 (24.9)	724 (75.1)	295 (30.6)	669 (69.4)
*χ*^*2*^	18.46^***^		14.04^**^		622.33^***^		78.17^***^		66.86^***^		171.42^***^	
**Social capital**	40.9 ± 14.6	42.4 ± 14.4	42.8 ± 15.3	41.4 ± 14.0	39.5 ± 14.2	46.0 ± 14.1	38.5 ± 14.2	44.8 ± 14.1	41.1 ± 14.1	42.3 ± 14.6	38.7 ± 13.6	44.6 ± 14.7
*t*	3.29^**^		−3.05^**^		15.48^***^		15.50^***^		2.59^*^		14.49^***^	
**HRQoL**	75.7 ± 12.6	78.0 ± 12.3	77.6 ± 12.6	77.1 ± 12.3	74.1 ± 12.3	82.7 ± 10.6	74.1 ± 12.4	80.0 ± 11.8	76.8 ± 12.2	77.5 ± 12.5	74.1 ± 11.9	80.0 ± 12.2
*t*	6.03^***^		−1.21		25.83^***^		17.15^***^		1.83		17.13^***^	

Continuous variables are presented as mean and standard deviation (*SD*), categorical variables are presented as frequency (*n*) and percentage (%)

***p < 0.001, **p < 0.01, *p < 0.05

### SEM results

CFA evaluated the measurement model for the latent variables: SES, social capital, and HRQoL. Model fit statistics for CFA indicated a good model fit of the measurement model with the following values: SRMR = 0.0695, RMSEA = 0.08, GFI = 0.96, CFI = 1.00, NFI = 0.95. Also, three latent variables showed good internal consistency reliability in this sample. The Cronbach’s coefficient alpha of PSCS-16 and WHOQOL-Old were 0.965 and 0.864, respectively (supplementary Table 1).

**Fig. 1:**
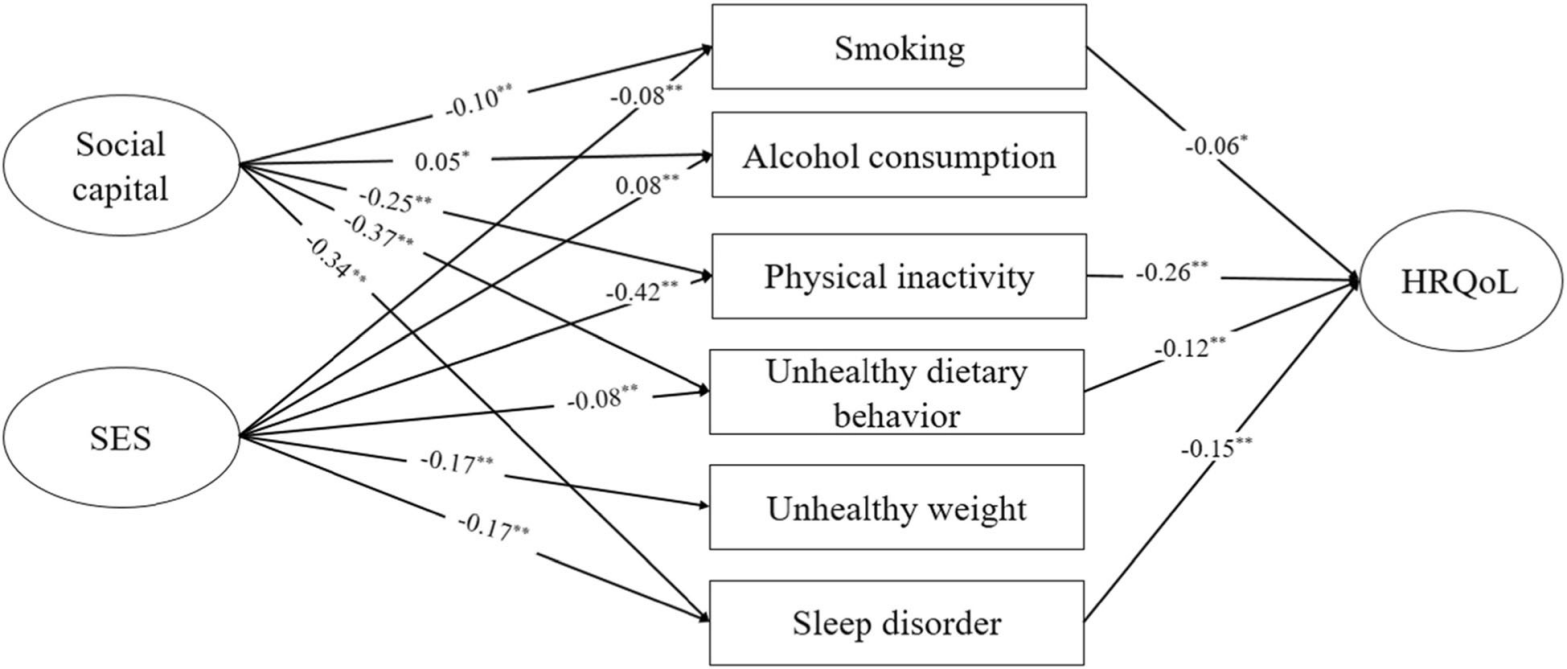
Structural model of associations between SES, social capital, health risk behaviors, and HRQoL. Note: numbers in the figure represent standardized path coefficients. Model fit statistics: SRMR = 0.0795, RMSEA = 0.07, GFI = 0.95, CFI = 0.91, NFI = 0.91. **p < 0.01, *p < 0.05

Similar to the measurement model, the model fit statistics suggested that the final SEM model fits our data well with the following values: SRMR = 0.0795, RMSEA = 0.07, GFI = 0.95, CFI = 0.91, NFI = 0.91. Figure 1 demonstrates the results of structural modeling. Social capital was positively associated with alcohol consumption (*β* = 0.05, *p* < 0.05) and negatively associated with smoking (*β* = − 0.10, *p* < 0.001), physical inactivity (*β* = − 0.25, *p* < 0.001), unhealthy dietary behavior (*β* = − 0.37, *p* < 0.001), and sleep disorder (*β* = − 0.34, *p* < 0.001). SES was positively associated with alcohol consumption (*β* = 0.08, *p* < 0.001) and negatively associated with smoking (*β* = − 0.08, *p* < 0.001), physical inactivity (*β* = − 0.42, *p* < 0.001), unhealthy dietary behavior (*β* = − 0.08, *p* < 0.001), unhealthy weight (*β* = − 0.17, *p* < 0.001), and sleep disorder (*β* = − 0.17, *p* < 0.001). In addition, smoking (*β* = − 0.06, *p* < 0.05), physical inactivity (*β* = − 0.26, *p* < 0.001), unhealthy dietary behavior (*β* = − 0.12, *p* < 0.001), and sleep disorder (*β* = − 0.15, *p* < 0.001) negatively associated with HRQoL.

## Discussion

Chinese elderly are undergoing unoptimistic health condition. Advocating healthy behavioral lifestyles and promoting elderly health have becoming a noteworthy topic in China [2]. In this study, we investigated six health risk behaviors of the Chinese elderly: smoking, alcohol consumption, physical inactivity, unhealthy dietary behavior, unhealthy weight, and sleep disorder. SEM results clarified the relationship between social capital, SES, different health risk behavior, and HRQoL.

In this study, the proportion of six health risk behaviors varied from 31.8 to 62.5%. Thirty-five percent of the participants reported smoking, which is much higher than the Chinese Center for Disease Control and Prevention reported in adults aged 15 years and older (26.6%) [33]. We found that 64.3 and 45.0% of the participants had physical inactivity and unhealthy dietary behavior, which was obviously at a high-risk level. As expected, the results of univariate analysis and SEM analysis indicated significant correlation between smoking, physical inactivity, unhealthy dietary behavior, sleep disorder, and poorer HRQoL. No significant correlations were found between alcohol consumption, unhealthy weight, and HRQoL. Thus, targeting appropriate behavioral interventions regarding these health risk behaviors could be helpful for elderly health promotion in China.

This study found that social capital negatively associated with smoking, physical inactivity, unhealthy dietary behavior, and sleep disorder. This is in agreement with previous studies [8–10, 16, 34]. We also revealed significant correlation between higher social capital and alcohol consumption. This fits with Chuang et al.’s [35] finding in Taiwan that social participation was positively associated with drinking in both male and female adults. In China, drinking is often regarded as a custom in social intercourse. Individuals with higher social capital are more socially active and have more opportunities for drinking. However, our finding was contrary to several previous studies conducted in Chinese population [20, 36]. Yuan [20] noted that social trust in community-level was a protective factor for Chinese elderly’s drinking behavior. Gao et al. [36] reported that higher individual-level social capital may protect against hazardous drinking among Chinese rural-urban migrant workers. Further studies are needed to understand the relationship between social capital and drinking behavior in Chinese society.

The results of SEM analysis generally confirmed the mediating role of health risk behaviors on the relationship between social capital and HRQoL among Chinese older adults. Social capital was detected to indirectly associate with the elderly’s HRQoL through the four health risk behaviors (*β* = 0.1664). The results were in consistent with Han et al.’s study [37] in Chinese adult residents. However, Poortinga et al.’s study [15] in English adult population aged 16 and over did not find the mediating role of health behaviors. The inconsistent results might be due to differences in study population. Generally, social capital played potential protective roles in improving HRQoL and decreasing some health risk behaviors. Thus, social capital should be considered as an important health component of elderly people and needs to be strengthened in a targeted way.

In this study, higher SES was negatively correlated with smoking, physical inactivity, unhealthy dietary behavior, unhealthy weight, and sleep disorder. We also found that SES positively correlated with the elderly’s HRQoL through four health risk behaviors (*β* = 0.1491). The findings are in consistent with previous research in Chinese adult residents [38, 39]. Thus, elderly people with low SES should be focused when it comes to health promotion and behavioral intervention in China.

This study also found positive correlation between SES and alcohol consumption, that is, elderly people with higher SES had higher proportion of alcohol consumption than those with lower SES. It can be seen that higher social capital and higher SES are both associated with the occurrence of drinking behavior in elderly people. The different result between drinking behavior and other health risk behaviors might be due to that most Chinese people considered drinking as a need for social communication and business engagement.

Chinese government implemented “Elderly Health Promotion Action” as part of the “Healthy China Action (2019-2030)” [2]. Improving elderly health requires an insight into the factors related to the health of Chinese older adult. This study reinforced the important role (direct and indirect) of health risk behaviors in elderly health, as well as the relationship between social capital, SES and HRQoL in Chinese older adults.

Several potential limitations should be mentioned regarding this study. Firstly, convenience sampling was used to recruit participants from both offline and online sources, and this study did not consider underlying diseases and medication uses of the participants, which may cause sampling bias. Secondly, indicators applied in this study were obtained through participants’ self-report, recall bias due to false or inaccurate responses could have played a role in our results. Thirdly, the cross-sectional nature of this study may be considered a weakness, as no causal inferences can be drawn from the results. For example, it may well be that poor health leads to lower social capital instead of the other way around. Longitudinal studies could provide more definite information on the possible causal pathways [40]. Fourthly, education level and family income level were included in the SEM analysis as ordered variable which may have potential effect on estimating results. Despite these limitations, this study is the first to explore the mediating role of different health risk behavior on the relationship between social capital, SES, and HRQoL in Chinese older adults. The results might be a valuable references for the implementation of current “Elderly Health Promotion Action” [2] and future relevant research.

## Conclusion

Chinese older adults demonstrated a high incidence of health risk behaviors, especially for physical inactivity (62.5%) and unhealthy dietary behavior (45.7%). Smoking, physical inactivity, unhealthy dietary behavior, and sleep disorder negatively associated with the elderly’s HRQoL. Both social capital and SES were found to be correlated with the elderly’s HRQoL, as well as with the health risk behaviors. Health risk behaviors played potential mediating role on the relationship between social capital, SES, and HRQoL in Chinese older adults. It is necessary to develop targeted intervention towards social capital and different health risk behaviors so as to improve elderly health. Moreover, the focus of policy on elderly people with different SES might make sense.

## Supplementary information

**Additional file 1: Supplementary figure 1.** Conceptual framework. **Supplementary table 1** Internal consistency and confirmatory factor analysis (CFA) results.

## Data Availability

The datasets generated during and/or analyzed during the current study are available from the corresponding authors on reasonable request.

## Abbreviations

BMI: Body Mass Index
CFA: Confirmatory Factor Analysis
HRQoL: Health-related Quality of Life
PSCS: Personal Social Capital Scale
*SD*: Standard Deviations
SEM: Structural Equation Model
SES: Socioeconomic Status
WHO: World Health Organization
