# Supplementary material for: Socioeconomic status, social capital, health risk behaviors, and health-related quality of life among Chinese older adults
Source: Health Qual Life Outcomes. 2020 Aug 28;18:291. doi: 10.1186/s12955-020-01540-8 (PMC7456043; doi:10.1186/s12955-020-01540-8)
Supplement: Supplementary file 1 — Additional file 1: Supplementary figure 1. Conceptual framework. Supplementary table 1 Internal consistency and confirmatory factor analysis (CFA) results. [file 12955_2020_1540_MOESM1_ESM.docx]

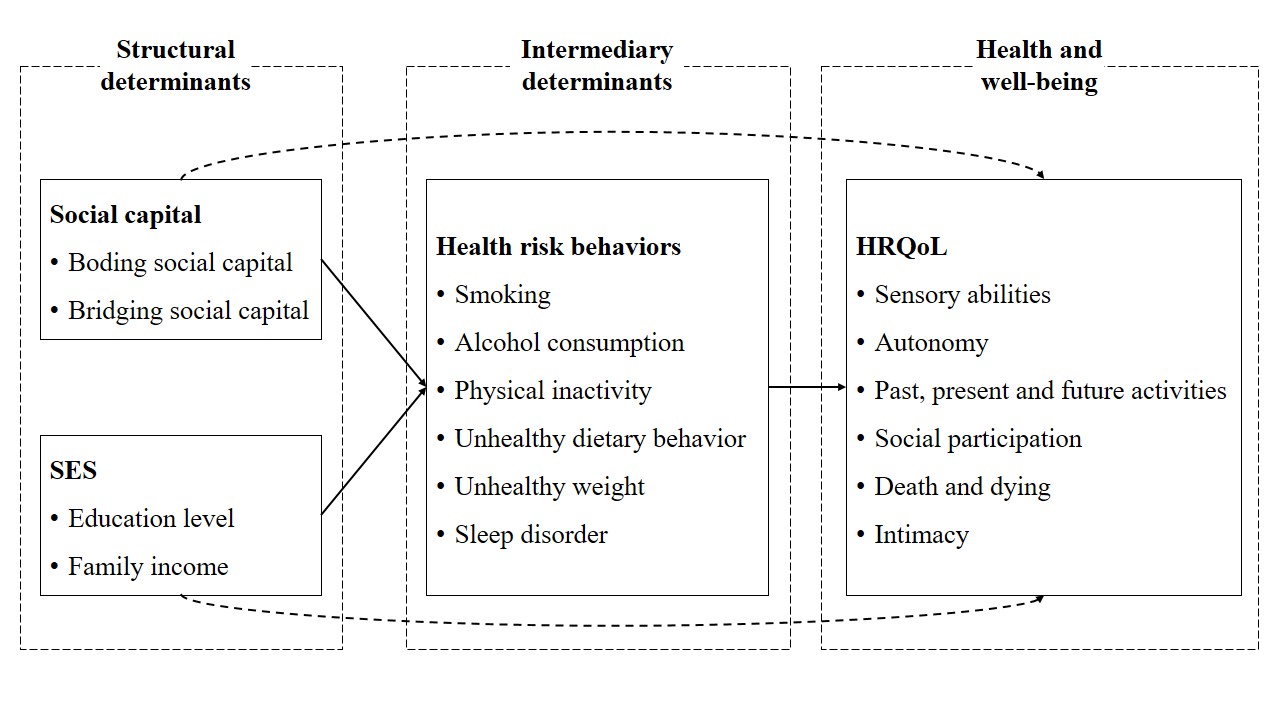


## Supplementary figure 1. Conceptual framework

Note: variables in rectangles represent manifest variables, and variables in ovals denote latent variables. Direct effects are indicated by solid lines. Indirect effects are indicated by dashed lines.

## Supplementary table 1. Internal consistency and confirmatory factor analysis (CFA) results.

| Latent construct | Manifest variable | Cronbach’s alpha | CFA | |
| --- | --- | --- | --- | --- |
|  |  |  | Standardized  factor loading | *p*-value |
| SES | Education level | - | 0.60 | <0.001 |
|  | Family income |  | 0.46 | <0.001 |
| Social capital | Bonding social capital | 0.965 | 0.98 | <0.001 |
|  | Bridging social capital |  | 0.78 | <0.001 |
| HRQoL | Sensory abilities | 0.864 | 0.18 | <0.001 |
|  | Autonomy |  | 0.86 | <0.001 |
|  | Past, present and future activities |  | 0.88 | <0.001 |
|  | Social participation |  | 0.86 | <0.001 |
|  | Death and dying |  | 0.09 | <0.001 |
|  | Intimacy |  | 0.63 | <0.001 |

Model fit statistics for CFA: SRMR = 0.0695, RMSEA = 0.08, GFI= 0.96, CFI= 1.00, NFI= 0.95.
